# The Impact of Radiation on an Unusual Case of Omental Epithelioid Angiosarcoma

**DOI:** 10.1155/2015/849059

**Published:** 2015-07-28

**Authors:** Sumana Narayanan, Mitchell Parker, Jonathan Shayo, Min Zheng, Theodore Matulewicz, Glenn Parker

**Affiliations:** ^1^Rutgers Robert Wood Johnson University Hospital, New Brunswick, NJ 08903, USA; ^2^Monmouth University, West Long Branch, NJ 07764, USA; ^3^Sackler School of Medicine, Tel Aviv University, 63305 Tel Aviv, Israel; ^4^Jersey Shore University Medical Center, Neptune, NJ 07753, USA

## Abstract

Epithelioid angiosarcoma is a rare high-grade tumor with irregular vascular morphology. We report an unusual case of intra-abdominal epithelioid angiosarcoma affecting the omentum and peritoneal surfaces resulting in significant hemorrhagic and inflammatory changes. As in other cases of this tumor this patient had previously undergone radiation treatment for a history of cervical cancer.

## 1. Introduction

Epithelioid angiosarcoma is an uncommon tumor that can present in a variety of locations and occurs with higher frequency in patients who have had exposure to ionizing radiation. The following is a case of a patient who developed epithelioid angiosarcoma in the omentum after radiation treatment for cervical cancer.

## 2. Case Report

**Figure 1:**
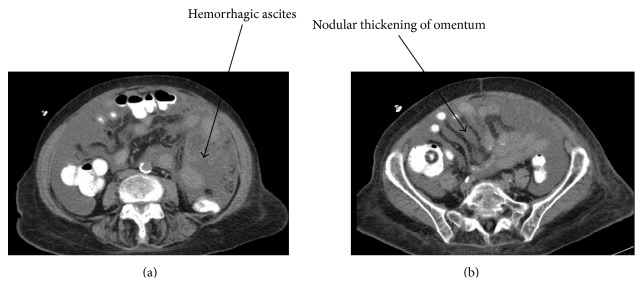
(a) Axial CAT scan images of extensive hemorrhagic ascites and (b) nodular thickening of the omentum and peritoneal surfaces.

A 77-year-old woman with a history of cervical cancer and radiation treatment presented with fecal incontinence as well as abdominal distension and discomfort after eating. She appeared thin with a softly distended abdomen but physical exam was otherwise unremarkable. Her past medical history also included hypertension, irritable bowel syndrome, left inguinal hernia repair, and a gastric ulcer. A colonoscopy revealed rectal stricture. CAT scan (Figure 1) noted a large amount of ascites as well as an ill-defined nodular thickening in the omentum and thickening of the peritoneal surfaces. Surgical intervention appeared to be the best option due to the patient's worsening diarrhea and CAT scan results.

The patient was taken to the operating room and a midline laparotomy incision was made. Upon entering the abdomen, two liters of ascitic fluid was identified. The small and large intestine were found to have diffuse hemorrhagic inflammatory changes as well as a bleeding and friable omentum. Omentectomy was performed and upon sending a frozen section, the patient was thought to have a high-grade adenocarcinoma. An ileostomy was then created to divert away from the strictured rectum. The patient had a postoperative course complicated by an ileus, aspiration pneumonia, and subsequent respiratory insufficiency, with ultimate referral to Palliative Care.

**Figure 2:**
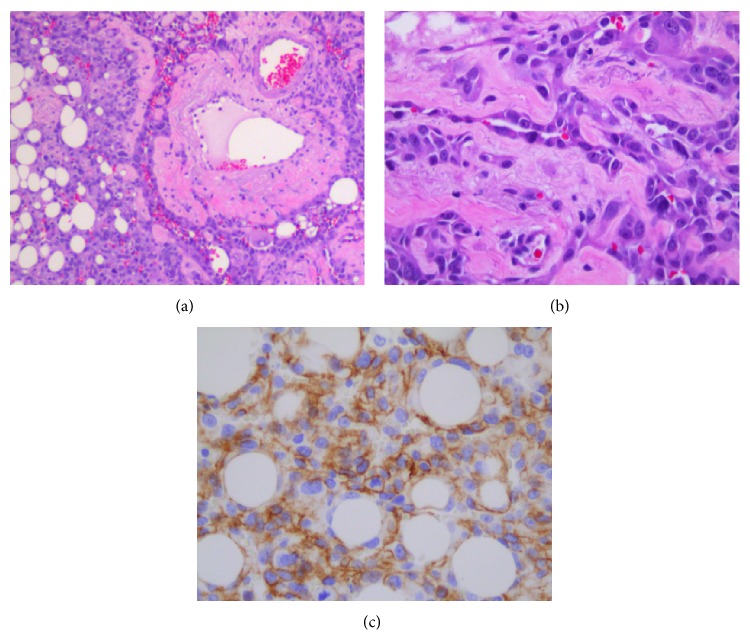
Pathologic analysis of omental epithelioid angiosarcoma. (a) H&E 100x: vascular proliferation replacing omental adipose tissue, (b) H&E 400x: tumor cells form complex vascular channels, and (c) CD31 400x: tumor cells expressing vascular marker CD31.

Pathologic analysis (Figure 2) of the omentum revealed a high-grade malignant epithelioid angiosarcoma, an extremely rare tumor. The tumor specimen appeared to have classic disorganized vascular architecture on microscopic examination and stained positive for Vimentin and p53, as well as for the vascular marker CD31.

## 3. Discussion

Epithelioid angiosarcoma is a very rare, highly vascular tumor with few published reports in the literature. These sarcomas arise from a wide variety of anatomic locations with most reported cases occurring in the axial skeleton and in soft tissues [1–3]. They also appear to have a predilection towards organs and tissues with higher vascularity and lymphatic supply as well as those comprised of large numbers of endothelial cells such as the thyroid and adrenal endocrine glands [1, 4].

Typically, epithelioid angiosarcomas have characteristic pathologic features such as irregular vascular channels with adjacent atypical pleomorphic epithelioid cells [5]. These tumors contain many mitotic figures as well as nuclear atypia and patchy areas of hemorrhagic necrosis [1, 5, 6]. Epithelioid angiosarcomas stain strongly positive for Vimentin and in most cases are immunoreactive for vascular markers CD31, CD34, and VEGFR-3 and Factor VIII [1, 7–9]. CD31 appears to be the most reliable and sensitive marker for these angiosarcomas [7]. These tumors are often confused with carcinomas because of their structural similarities but may be distinguished because these sarcomas often coexpress epithelial antigens such as EMA, Cam5.2, and AE1/3 [6].

Intra-abdominal epithelioid angiosarcomas are an extremely uncommon entity. One case series by Allison et al. examined the presentation and outcomes of patients with angiosarcomas of the gastrointestinal (GI) tract. These patients, in majority, presented with hematochezia or melena and anemia. They underwent tumor resection and were found to have high-grade or metastatic angiosarcoma. Only one patient's tumor could be completely resected and after receiving chemotherapy and radiation, the patient survived for 27 months. All others continued to have GI bleeding from metastatic implants and eventually expired from their disease [7]. Epithelioid angiosarcoma of the peritoneal surfaces and omentum appear to be even less common than cases that involve the gastrointestinal tract. One such case was discovered upon laparotomy with copious intra-abdominal hemorrhage and extensive friable, nodular implants which continued to bleed upon manipulation [10].

Other intra-abdominal cases of this highly malignant sarcoma have been reported in patients who have received prior radiation. A case of small intestinal epithelioid angiosarcoma was identified in a patient with a history of thirty years of occupational exposure to radiation and polyvinyl chloride and developed gastrointestinal bleeding [11]. This patient was readmitted one month after resection with malignant ascites and peritoneal studding and expired from respiratory failure soon after reoperation [11]. Radiation-Associated Angiosarcoma (RAAS) occurs most commonly after treatment for breast cancer and is on average diagnosed seven years after radiation therapy [8]. Sarcomas associated with radiation appear to have a worse prognosis compared with sporadic soft tissue sarcomas (17–41% 5-year survival) [8].

Factors additionally noted to influence the development of angiosarcomas include toxic chemical exposure, Thorotrast contrast media, Dacron vascular grafts, arteriovenous fistulae, and chronic lymphedema [9]. Hepatic angiosarcoma development in particular has been found in a number of studies to correlate with exposure to arsenic, vinyl chloride monomer, androgenic anabolic steroids, and Thorotrast as well as radiation [12, 13]. A few case reports of intra-abdominal angiosarcoma have also been noted to occur secondary to foreign body inflammatory reaction often due to gauze left from previous surgery with subsequent formation of a fibrous capsule over the foreign body [14–16].

Intraperitoneal epithelioid angiosarcoma is a rare entity. It often presents with severe bleeding secondary to diffuse spread and friability of nodules. Radiation exposure significantly increases the likelihood of development of this sarcoma. The majority of these tumors are diagnosed late and have a poor prognosis as most patients have metastatic disease or diffuse peritoneal implants at the time of discovery.
